# Nitrate-Functionalized poly(ε-Caprolactone) Small-Diameter Vascular Grafts Enhance Vascular Regeneration *via* Sustained Release of Nitric Oxide

**DOI:** 10.3389/fbioe.2021.770121

**Published:** 2021-11-30

**Authors:** Sen Yang, Xueni Zheng, Meng Qian, He Wang, Fei Wang, Yongzhen Wei, Adam C. Midgley, Ju He, Hongyan Tian, Qiang Zhao

**Affiliations:** ^1^ Department of Peripheral Vascular Disease, First Affiliated Hospital of Xi’an Jiaotong University, Xi’an, China; ^2^ Department of Vascular Surgery, Tianjin First Central Hospital, Nankai University, Tianjin, China; ^3^ State Key Laboratory of Medicinal Chemical Biology, College of Life Sciences, Nankai University, Tianjin, China; ^4^ Key Laboratory of Bioactive Materials (Ministry of Education), College of Life Sciences, Nankai University, Tianjin, China; ^5^ Zhengzhou Cardiovascular Hospital and 7th People’s Hospital of Zhengzhou, Zhengzhou, China

**Keywords:** small-diameter vascular grafts, nitric oxide, vascular regeneration, vascular progenitor cells (VPCs), vascular calcification

## Abstract

Artificial small-diameter vascular grafts (SDVG) fabricated from synthetic biodegradable polymers, such as poly(ε-caprolactone) (PCL), exhibit beneficial mechanical properties but are often faced with issues impacting their long-term graft success. Nitric oxide (NO) is an important physiological gasotransmitter with multiple roles in orchestrating vascular tissue function and regeneration. We fabricated a functional vascular graft by electrospinning of nitrate-functionalized poly(ε-caprolactone) that could release NO in a sustained manner via stepwise biotransformation *in vivo*. Nitrate-functionalized SDVG (PCL/NO) maintained patency following abdominal arterial replacement in rats. PCL/NO promoted cell infiltration at 3-months post-transplantation. In contrast, unmodified PCL SDVG showed slow cell in-growth and increased incidence of neointima formation. PCL/NO demonstrated improved endothelial cell (EC) alignment and luminal coverage, and more defined vascular smooth muscle cell (VSMC) layer, compared to unmodified PCL SDVG. In addition, release of NO stimulated Sca-1^+^ vascular progenitor cells (VPCs) to differentiate and contribute to rapid luminal endothelialization. Furthermore, PCL/NO inhibited the differentiation of VPCs into osteopontin-positive cells, thereby preventing vascular calcification. Overall, PCL/NO demonstrated enhanced cell ingrowth, EC monolayer formation and VSMC layer regeneration; whilst inhibiting calcified plaque formation. Our results suggested that PCL/NO could serve as promising candidates for improved and long-term success of SDVG implants.

## Introduction

Cardiovascular disease (CVD) is the leading cause of global mortality (Roth et al., 2015; Virani et al., 2021). CVDs are frequently associated with damaged vasculature, functional inadequacies of blood vessels, and peripheral arterial diseases. Surgical replacement of the dysfunctional blood vessels with autologous vessels, such as saphenous veins and radial internal mammary arteries, are considered the gold-standard option for intervention. However, limited availability of suitable donor vessels, donor site co-morbidities, infection risk, and prevalence of long-term graft complications necessitate the development of alternative options. Synthetic polymer vascular grafts, including expanded polytetrafluoroethylene (ePTFE; Teflon/GORE-TEX) and polyethylene terephthalate (PET; Dacron) tubular prosthetics, are clinically employed as large diameter vascular grafts, but fail to perform adequately when used as small diameter vascular grafts (SDVG; ≤ 6 mm). Explanations for high failure rates are largely related to immunogenicity, poor hemocompatibility of the materials, and lack of bioactivity; often resulting in thrombosis, intimal hyperplasia, atherosclerosis, calcification, infection, and subpar long-term patency (Greenwald and Berry, 2000; Zilla et al., 2007; Hadinata et al., 2009). Thus, progression in the bioengineering of artificial alternatives for SDVGs is driven by urgent necessity.

The use of polycaprolactone (PCL) to manufacture “off-the-shelf” vascular grafts yields biocompatible artificial SDVGs that exhibit patency and mechanical properties capable of withstanding arterial blood flow pressure (de Valence et al., 2012). In combination with the versatile technique of electrospinning, PCL can be processed into fibrous extracellular matrix (ECM)-like materials with fiber and pore diameters ranging from the micrometer to nanometer scale, which enables the tuning of structural scaffold mechanical properties and to facilitate cell attachment, migration, and bioactive factor or nutrient exchange (Park et al., 2019). However, PCL vascular grafts have slow degradation rates, poor hydrophilicity, and low bioactivity (Pektok et al., 2008; Valence et al., 2013; Yang et al., 2019). Therefore, cell ingrowth, vascularization, and ECM deposition is hampered in long-term implantation. The timely regeneration of a functional endothelium provides an antithrombotic interface (Ren et al., 2015) and regulates the formation of organized vascular smooth muscle cell (VSMC) layers during vessel remodeling (McCallinhart et al., 2020). Functionalization of PCL grafts can help achieve antithrombotic actions (Yao et al., 2014; Wei et al., 2019) until a functional endothelium has formed on the graft lumen. It is the consensus that endothelial cells (ECs) have a limited migration ability from anastomotic sites (Zheng et al., 2012). Thus, augmenting PCL SDVG bioactivity is necessary to achieve rapid re-endothelization and to limit occurrence of vascular complications.

Nitric oxide (NO) is a gasotransmitter and signaling molecule, endogenously produced by the endothelium. NO is essential in maintaining cardiovascular homeostasis (Yang et al., 2018) and dysfunctions in NO signaling is often an associated-complication of CVD pathologies (Erdmann et al., 2013). NO-based interventions were shown to attenuate myocardial infarction (Rassaf et al., 2014; Zhu et al., 2021), thrombus formation (Heusch, 2015; Heusch and Gersh, 2017; Yang et al., 2020a), and convey immunomodulatory and cardioprotective effects (Bogdan, 2001; Chouchani et al., 2013). NO release or generation mechanisms have been incorporated into multiple cardiovascular implants, including stents (Zhang et al., 2019; Yang et al., 2020b) and vascular grafts (Wang et al., 2015), with demonstratable benefits to the promotion of angiogenesis, re-endothelialization, and vascular tissue regeneration (Yang et al., 2015; Li et al., 2018; Kabirian et al., 2019). In addition to the l-arginine derived NO, generation of NO can be achieved from biotransformation of endogenous NO-donors, such as S-nitrosothiols and nitrates present in blood and tissues (Lundberg et al., 2008; Panesar, 2008; Lundberg et al., 2009; Qian et al., 2021). However, the bioavailability of circulating endogenous NO donors at any one time is finite and with the added complications of reactivity, short half-life, labile nature, and instability of NO (Nichols et al., 2012; Midgley et al., 2020), achieving long-term controlled release at efficacious concentrations by NO-generating/NO-donor functionalised vascular grafts remains a major clinical challenge.

Accumulating evidence have implicated NO in diverse regulatory effects on progenitor and stem cells, including impacting paracrine secretion patterns, and the production of growth factors and exosomes. (Bonafè et al., 2015; Midgley et al., 2020). In addition, endothelial nitric oxide synthase (eNOS) was implicated in the mobilization of stem and progenitor cells from cardiovascular niches (Aicher et al., 2003). In murine vessels, stem cell antigen-1 (Sca-1) expressing vascular progenitor cells (VPCs) in the adventitia were shown to migrate to the intima and contribute to vascular remodeling (Hu et al., 2004; Covas et al., 2005; Ingram et al., 2005). Sca-1^+^ VPCs from the media of mouse abdominal aorta could differentiate into ECs or VSMCs in response to angiogenic growth factors *in vitro* (Sainz et al., 2006). Under stable physiological conditions *in vivo* Sca-1+ VPCs differentiate into a balanced ratio of EC:VSMC, which becomes dysregulated during vascular pathogenesis (Torsney and Xu, 2011). Cardiac resident Sca-1+ progenitor cells were implicated in contributing to cardiac vasculature regeneration, with involvement in EC expansion after myocardial infarction (Vagnozzi et al., 2018). Therefore, resident Sca-1^+^ VPCs contribution to ECs and VSMCs during vascular tissue repair may be key events important in limiting the occurrence of intimal hyperplasia. Whether the vasoprotective effect of NO during vascular remodeling is associated with the modulation and participation of resident Sca-1^+^ VPCs, is currently unknown.

In this study, we designed nitrate-functionalized vascular grafts based on a design concept that utilizes a blend of low-molecular weight nitrate-functionalized PCL polymers with high-molecular weight PCL. PCL/NO SDVGs were first tested for mechanical properties. Consistent and controlled long-term release of NO was modelled *in vitro* by incubation of grafts with enzymes to mimic *in vivo* biotransformation cascades. Rat abdominal aorta replacement models were used to assess the performance of implanted PCL/NO SDVG and to demonstrate the beneficial effects of sustained local NO delivery in the regeneration of vascular tissues. The capacity for PCL/NO SDVGs to induce Sca-1^+^ VPC recruitment and direct their differentiation into vascular cells during vascular regeneration was also assessed. The results of this study support the intention to establish NO-functionalized graft fabrication strategies for substantial clinical viability and improvement of long-term SDVG implant success.

## Materials and Methods

### Fabrication of Nitrate-Functionalized Vascular Grafts

Briefly, PCL (*M*_n_ 80,000) was mixed with PCL-ONO_2_ (*M*_n_ 2,000) at blending ratios of 9/1 (w/w), according to our previously published protocol (Zhu et al., 2021). The mixture was dissolved in mixed chloroform/methanol (5:1, v/v) by sufficient stirring to obtain homogeneous solution with a final concentration of 10% (w/v). Microfiber grafts were fabricated by electrospinning using a setup previously described with minor modification (Wei et al., 2019). Briefly, polymer solution was ejected at a continuous rate (2 ml/h) using a syringe pump through a stainless-steel needle (2 mm i.d.) and collected on a rotating stainless-steel mandrel. A high voltage (15 kV) was applied to the needle with a variable high-voltage power supplier. Electrospinning continued until scaffold wall thickness reached approximately 350 μm. The resulting scaffold was then removed from the mandrel and placed into a vacuum overnight to remove the residual solvent. Prior to use in experiments, the grafts were sterilized by immersion in 75% ethanol for 30 min, and then exposed to UV light overnight.

### Characterization and Composition

The surface morphology of electrospun mats was observed under a field emission scanning electron microscopy (SEM; Quanta 200, Czech Republic) at an accelerating voltage of 10 KV. The surface was sputter-coated with gold before observation. The surface chemistry was characterized using Fourier transform infrared spectroscopy (FTIR) and at a single attenuated total reflectance (ATR) mode (Bio-Rad FTS 6000 Spectrometer: spectral resolution, 8 cm^−1^). The PCL mats were subjected to energy dispersive X-ray spectroscopy (EDS) using a Quanta FEI 650 device (Quanta FEI). The quantification of elements (C, N, O) in the region struck by the rapid electron were determined graphically.

### Mechanical Tests

The mechanical properties of the scaffolds were assessed by a tensile-testing machine with a load capacity of 1 kN (Instron). Samples with 6.28 mm width, 350 μm thickness, and 2 cm length in each scaffold group (*n* = 3) were prepared. The inter-clamp distance was set as 1 cm, and then, samples were pulled longitudinally at a rate of 10 mm/min until rupture. The stress-strain curves of the scaffolds were recorded. The Young’s modulus was calculated based on the slope of the stress-strain curve in the elastic region. Burst pressure was measured by filling a graft segment (3 cm length) with soft paraffin (Vaseline) whilst clamping one end and hermetically sealing the other with a vascular catheter. A constant filling rate of 0.1 ml/min was applied, and the filling pressure was recorded until the graft wall burst.

### NO Release From PCL/NO Grafts

The NO releasing profile was determined by 3-Amino, 4-aminomethyl-2′, 7′-difluorescein, diacetate (DAF-FM) probe (Beyotime, China) according to the manufacturer’s protocol. Briefly, 6 mg of nitrate-functionalized materials were put into 2 ml of PBS buffer (pH 7.4) containing DAF-FM (5 μM), and muscular homogenate or peritoneal fluid were added at certain concentration to act as catalyst for the NO generation, respectively. After pre-determined time interval, solution was transferred into 96-well plates, and the fluorescence intensity was measured by a microplate reader (Synergy 4-BioTek, USA).

### *In vitro* Nitrate Release

10 mg nitrate-functionalized materials were incubated in PBS buffer at 37°C. The production of nitrate was evaluated according to the Griess method using the Total Nitric Oxide Assay Kit (Beyotime Biotechnology, S0023). The optical density was measured at 540 nm, and the amount of nitrate was determined using sodium nitrate as a reference standard.

### Measurement of NO Generation From Nitrates

NaNO_3_ was dissolved into 2 ml of PBS, or the muscular homogenate or peritoneal fluid at a final concentration of 200 μM. Samples were placed on a shaker at 37°C and the NO generation was determined by DAF-FM probe. After pre-determined time interval, solution was transferred into 96-well plates, and the fluorescence intensity was measured by a microplate reader (Synergy 4-BioTek, USA).

### *In vivo* Implantation

The use of experimental animals was approved by the Animal Experiments Ethical Committee of Nankai University and carried out in conformity with the Guide for Care and Use of Laboratory Animals. The procedure was carried out as described previously. In brief, rats were anesthetized with chloral hydrate (300 mg/kg) by an intraperitoneal injection. Heparin (100 units/kg) was administered for anticoagulation by tail vein injection before surgery. A midline laparotomy incision was then performed, and the abdominal aorta was isolated, clamped, and transected. The tubular PCL grafts (2.0 mm in inner diameter and 1.0 cm in length) were sewn in an end-to-end fashion with 8–10 interrupted stitches using 9–0 monofilament nylon sutures (Yuan Hong, Shanghai, China). No anticoagulation drug was administered to the rats after surgery. At the predetermined time points (1 and 3 months), the patency and blood velocity of the grafts was visualized by high-resolution ultrasound (Vevo 2100 System, Canada) after the rats were anesthetized with isoflurane.

### Histological Analysis

After 3 months, animals were sacrificed by injection of overdose chloral hydrate. Subsequently, grafts were explanted, rinsed with saline, and cut into two parts from the middle. One part was sectioned into suture-site, quartile, and middle segments (1.5 mm) for frozen cross-sectioning. The other half was longitudinally cut into two pieces. One piece was first observed by stereomicroscope, and then for frozen longitudinal section. The other piece was processed for SEM examination.

Briefly, the samples were fixed with 2.5% glutaraldehyde overnight, and dehydrated in a sequence of ethanol solutions for 5 min each. After air-drying at room temperature, samples were mounted onto aluminium stubs and then sputter-coated with gold for SEM.

After embedded in OCT and snap-frozen in liquid nitrogen, the samples were cryo-sectioned to 6 mm in thickness. Subsequently, sections were stained with H&E (Legend) or von Kossa (Legend) using standard histological methods. Images were observed under an upright microscope using a 4×, 10× or 20× objective lens (Leica, Germany), photographed and then analysed using ImageJ software (1.8.0, NIH, Bethesda, MD, USA).

For immunofluorescent staining, the frozen sections were fixed in cold acetone for 10 min, air-dried, and rinsed once with 0.01 mM PBS. Then slides were incubated in 5% normal goat serum (Zhongshan Golden bridge Biotechnology, China) for 45 min at 4 C. For intracellular antigen staining, 0.1% Triton-PBS was used to permeate the membrane before incubation with serum. Then the sections were incubated with primary antibodies in PBS overnight at 4 C, followed by incubation with secondary antibody in PBS for 2 h at room temperature. The nuclei were counterstained with 4,6-diamidino-2-phenylindole (DAPI) containing mounting solution (DAPI Fluoromount G, Southern Biotech, UK).

Endothelial cell staining was performed using monoclonal mouse anti-CD31 (Abcam, 1:100) primary antibody. The smooth muscle cells were stained using rabbit anti-α-SMA (Abcam, 1:100) and mouse anti-smooth muscle myosin heavy chain (SM-MHC, Santa Cruz, 1:100) primary antibodies. Rabbit anti-Sca-1 (Abcam, 1:100) was used to visualize vascular progenitor cells. Goat anti-mouse IgG (1:200, Invitrogen) and goat anti-rabbit IgG (1:200, Invitrogen) were used as the secondary antibodies. Sections that were not incubated with primary antibodies were used as negative controls to assess and eliminate background staining. Immunohistochemistry images were captured by fluorescence microscope (Zeiss Axio Imager Z1, Germany) and analysed by ImageJ software (1.8.0 NIH). Endothelial coverage rate was calculated according to the following equation:

$$\text{Endothelial}\;\text{coverage}\left(\text{\%}\right)=\frac{\text{Length}\;\text{of}\;\text{CD}31\;\text{labelled}\;\text{endothelium}\left(\text{µm}\right)}{\text{Total}\;\text{length}\;\text{of}\;\text{longitudinal}\;\text{graft}\;\text{section}\left(\text{µm}\right)}\;\times 100$$

Four sections per sample and four samples per group were included to obtain the statistical results.

### Statistical Analysis

All quantitative *in vitro* results were obtained from at least three samples for analysis. All qualitative and quantitative *in vivo* results were obtained from at least four animal per experimental group. GraphPad Prism software v5.0 (San Diego, CA, USA) was used for statistical analysis. Student’s *t*-tests were used for comparisons of means between two groups. Comparisons of means among three or more groups were done by one-way ANOVA and for grouped data with two or more variables, two-way ANOVA was performed. Differences were considered significant at *p =<0.05 and **p = <0.01.

## Results

### Characterization of Nitrate-Functionalized PCL Vascular Grafts

**FIGURE 1:**
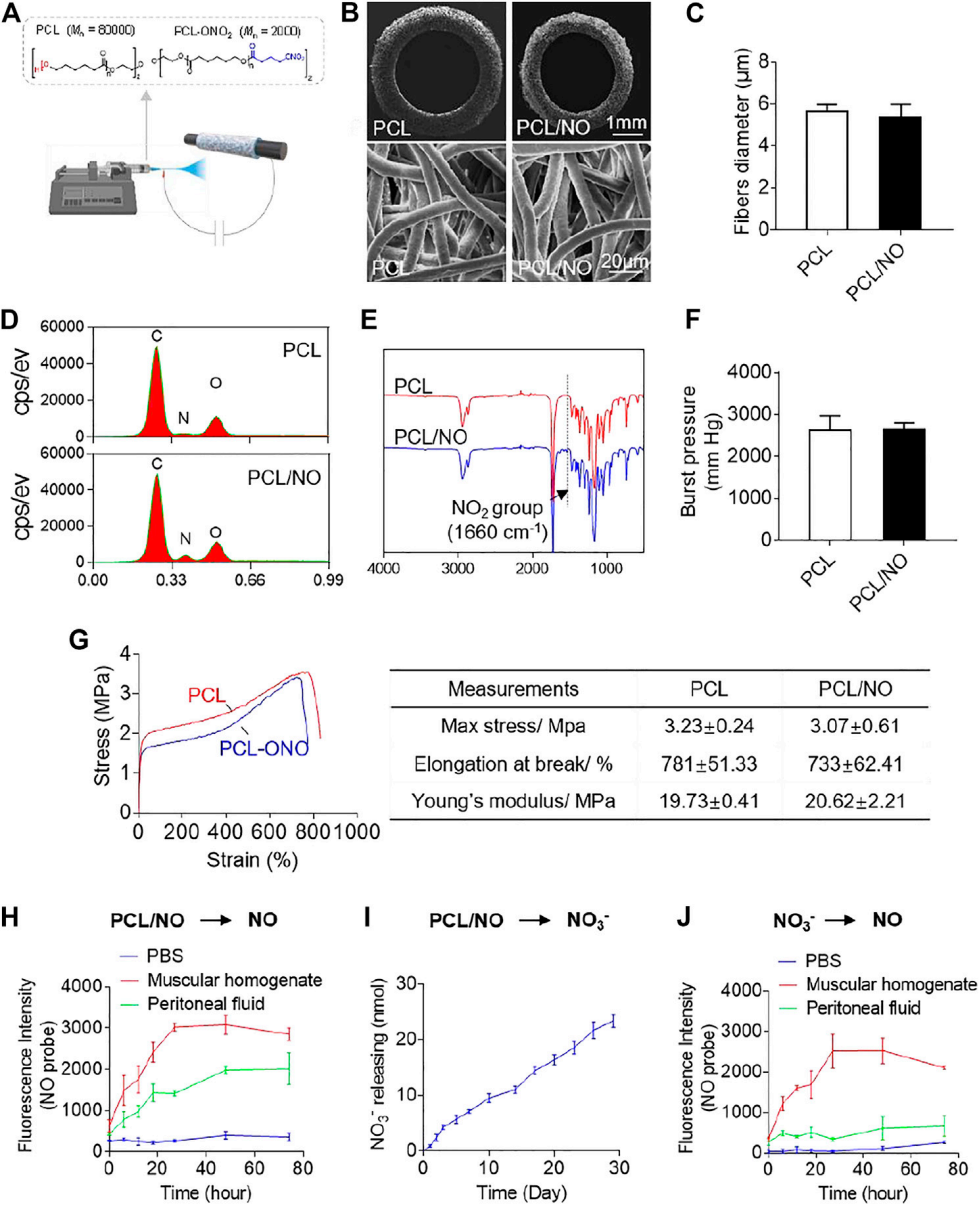
Fabrication and characterization of nitrate-functionalized SDVGs. **(A)** Schematic illustrating the PCL/NO SDVG synthesis by electrospinning of PCL blends. **(B)** SEM images of SDVG enface cross-sections (**top row**; scale bar = 1 mm) and surface networks of PCL microfibers (**bottom row**; scale bar = 20 µm). **(C)** Average PCL microfiber diameter in PCL and PCL/NO SDVGs. (D) EDS analysis of the SDVGs demonstrating an elevated nitrogen-peak in PCL/NO. (E) FTIR analysis of the SDVGs demonstrating the presence of NO2 groups (1,660 cm-1) in PCL/NO grafts. **(F)** The burst pressure of the SDVGs. **(G)** Representative stress-strain curves of the PCL and PCL/NO vascular grafts and the quantitative analysis of mechanical properties shown in the table alongside. Data presented as mean ± S.E.M. **(H)** NO generation from PCL/NO in the presence of muscular homogenate (20 mg/ml) or peritoneal fluid was detected by DAF-FM *in vitro*. **(I)** The long-term and slow accumulative release of nitrate and nitrite ions from SDVGs over 30 days in PBS buffer. **(J)** Reduction of NO3− into NO in the presence of muscular homogenate (20 mg/ml) or peritoneal fluid. Images and data are representative of *n* = 3 independent experiments.

Fabricated PCL and PCL/NO SDVGs were successfully prepared by electrospinning (Figure 1A) and visualized by SEM (Figure 1B). SDVGs exhibited homogenously distributed fibers within the graft. The tubular SDVGs had regular and uniform structure with inner diameters of 2 mm. The SDVGs possessed well-defined fiber morphology, and the average fiber diameter of PCL and PCL/NO SDVGs was 5.62 ± 0.36 μm and 5.36 ± 0.63 μm, respectively (Figure 1C). EDS analysis demonstrated an elevated nitrogen peak within the PCL/NO graft group, indicating the increased nitrogen content (-ONO_2_) (Figure 1D). Furthermore, FTIR verified incorporation of -ONO_2_ groups in the PCL/NO grafts, as evidenced by characteristic NO_2_ group peak (1,660 cm^−1^), which was only present in the PCL/NO graft group (Figure 1E). The assessed burst pressure of the PCL and PCL/NO grafts showed that there was no significant difference between the PCL and PCL/NO grafts. Both grafts demonstrated burst pressure values above the 1,600 mmHg threshold for withstanding arterial pressure (Figure 1F). Mechanical testing indicated that PCL/NO had a non-significant tendency to exhibit lower tensile strength and elongation at break than PCL, whereas calculated Young’s moduli of PCL/NO SDVGs also showed no significance difference compared to PCL (Figure 1G). The *in vitro* release of NO from nitrate-functionalized SDVGs was first evaluated by NO fluorescent probe (DAF), and generation of NO was detected in the PBS buffer with the addition of peritoneal fluid or muscular homogenate (Figure 1H), suggesting that the biotransformation is catalyzed by the relevant enzymes in the abdominal microenvironment where the graft was implanted. To get further insight into the transformation mechanism, the hydrolysis of nitrate-functionalized SDVGs in PBS was investigated. Nitrate ions (NO_3_^−^) were the main hydrolysis product, and the release of NO_3_^−^ demonstrated a sustained and linear accumulated release profile over 30 days (Figure 1I). The released NO_3_^−^ could be further reduced to NO under the catalysis of reductase present in the muscle instead of abdominal fluid (Figure 1J). These results suggested that the biotransformation of nitrate-functionalized PCL may proceed through two distinct pathways. First, it could be reduced directly into NO by the reductase in the peritoneal fluid, similar to the transformation of organic nitrates, such as GTN (Ferreira and Mochly-Rosen, 2012). Additionally, as a macromolecular nitrate, PCL-ONO_2_ undergoes non-enzymatic hydrolysis to release nitrate anion. Then, the generated nitrate anions are further reduced to release NO via the NO_3_→NO2→NO sequential pathway under the catalysis of reductase within muscular tissues (Zhu et al., 2021).

### *In vivo* Implantation and Performance of Nitrate-Functionalized PCL Vascular Grafts

**FIGURE 2:**
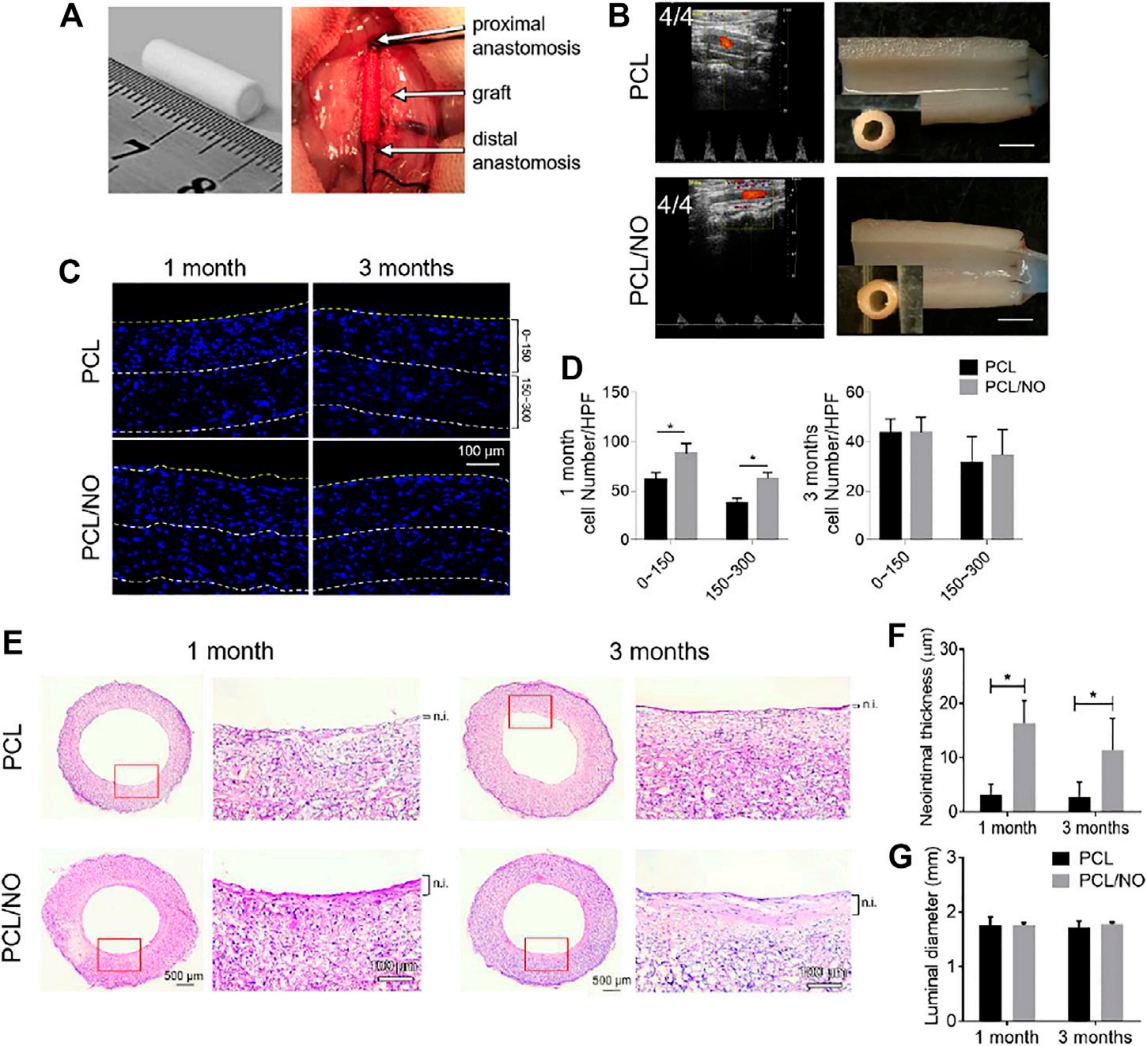
*In vivo* performance of SDVGs in abdominal aorta replacement rat models. **(A)** Photographic representation of fabricated grafts **(left)** and surgical implantation and anastomosis of SDVGs in rat abdominal aortae **(right)**. **(B)** High-resolution ultrasound imaging of implanted SDVGs at 3 months, patency rating inset **(left panels)**. Longitudinally cut transections of SDVGs and observation by stereomicroscope, inset panels are stereomicroscope images of enface cross-sections before transection, scale bars = 2 mm **(right panels)**. **(C)** DAPI stained sections of SDVGs at 1- and 3-months post-implantation to visualize cell infiltration at 0–150 μm and 150–300 µm graft depths. Yellow dashed line indicates the graft lumen surface, white dashed lines indicate 150 and 300 µm depths from the lumen surface. Scale bar = 100 µm. **(D)** The average cell number per high-powered field (HPF) within different SDVG depths at 1 month **(left)** and 3 months **(right)**. **(E)** H&E staining of enface SDVG sections. Scale bars = 500 µm. Red boxes indicate the view shown in zoomed images alongside each panel. Scale bars = 100 µm. The neointima (n.i.) is labeled alongside zoomed panels. Calculated **(F)** average neointimal thickness and **(G)** luminal diameter of PCL and PCL/NO SDVGs at 1- and 3-months post-implantation. Images and data are representative of *n = 4* independent experiments.

The fabricated SDVGs were assessed for 3-months *in vivo* performance using a rat abdominal artery replacement model (Figure 2A). After 3 months of implantation time, the patency rates of both SDVGs were 4/4, and excised grafts showed no sign of material rupture, structural deformation, or stenosis after 3 months of implantation time (Figure 2B). Immunohistochemistry staining of DAPI in SDVG enface cross-sections (Figure 2C) demonstrated a significantly enhanced cellular infiltration at 0–150 μm and 150–300 µm depths from the luminal surface by 1 month in PCL/NO SDVGs compared to PCL SDVGs, and by 3 months post-implantation the cell numbers within grafts were consistent amongst both grafts and at the assessed graft depths (Figure 2D). H&E staining showed that PCL SDVGs developed a thin neointimal layer at 1 and 3 months after implantation, whereas a thicker neointimal tissue layer was present in PCL/NO SDVGs at 1 and 3 months after implantation (Figure 2E). The average neointimal thickness of PCL/NO was significantly greater than that in the PCL group (Figure 2F), whereas luminal diameter (Figure 2G) remained unchnaged at 1- and 3-months in both graft groups. The results indicated that PCL/NO SDVGs promoted cell infiltration and accelerated neointimal formation whilst maintaining graft structure and performance.

### Nitrate-Functionalized Grafts Have Enhanced Endothelium Coverage

**FIGURE 3:**
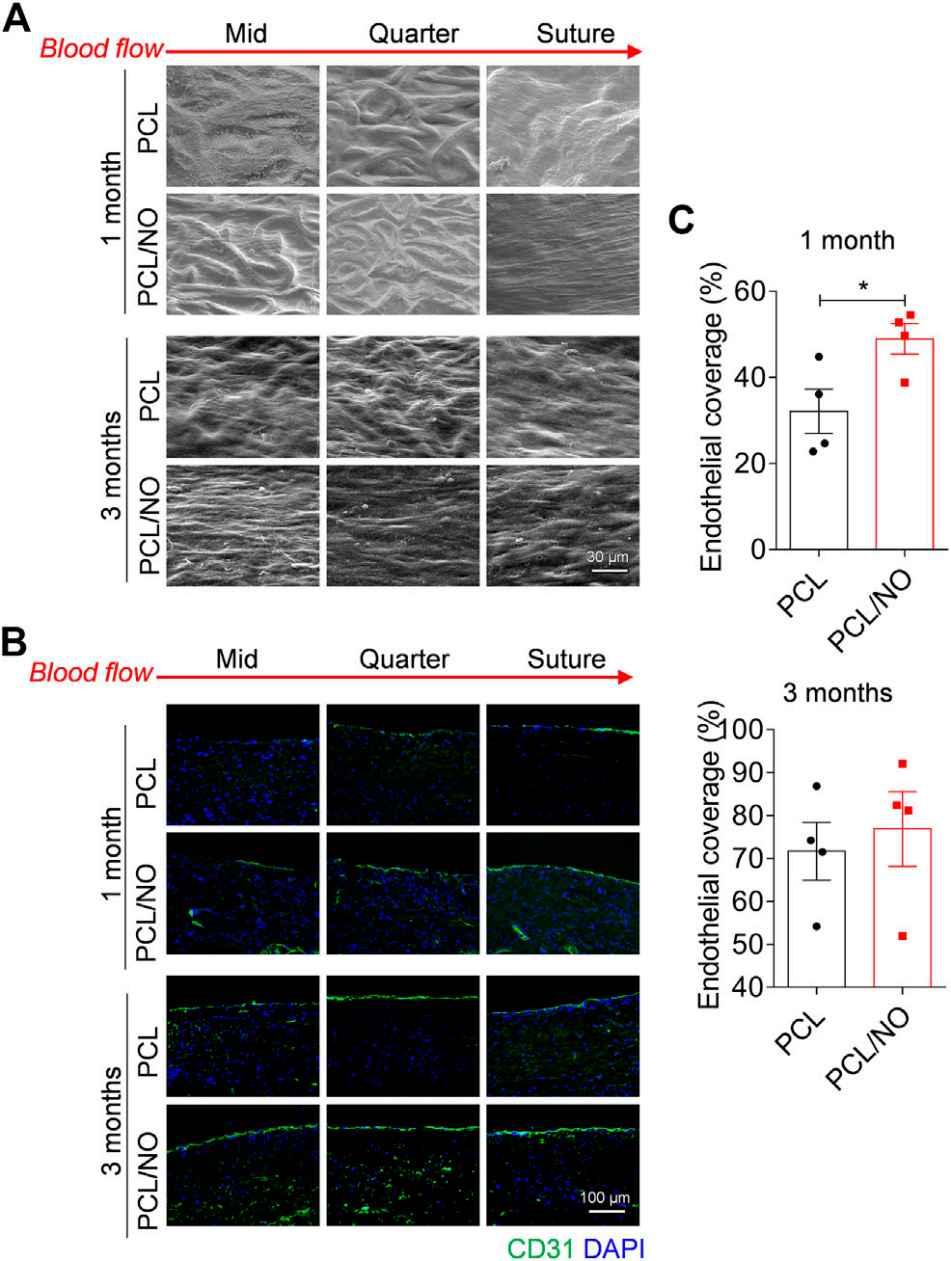
Sustained NO release from nitrate-functionalized SDVGs increases endothelialization. **(A)** SEM images of the luminal surface of the SDVGs at the mid-, quarter- and suture-site segments after 1 month and after 3 months (scale bars = 30 µm). Images orientated to blood flow direction. Red dashed boxes indicate location of zoomed inset images outline in red. **(B)** CD31 staining of endothelial cells within transverse sections of the SDVGs at the mid-, quarter- and suture-site segments after 1 month **(top rows)** and after 3 months **(bottom rows)**. Images orientated to blood flow direction. Scale bars = 100 µm. **(C)** Quantification of the average percentage of endothelial cell monolayer coverage of SDVG lumens at 1 month **(top)** and at 3 months **(bottom)**. Images and data are representative of *n* = 4 independent experiments.

To assess the degree of SDVG endothelialization, SEM was used to visualize endothelial coverage on the luminal surface (Figure 3A). At 1 month, PCL and PCL/NO demonstrated a degree of cell coverage; at the mid-, quarter-, and suture-sites. PCL microfibers could still be identified, but less so in the suture site segments of the lumens. At 3 months, PCL microfiber structures could be seen at the mid and quarter site of the PCL graft lumen, but EC coverage was evident and was improved at the suture site. The majority of the lumen sections in PCL/NO grafts exhibited ECs with tight cell junctions aligned to the blood flow direction, and individual PCL microfiber structures were difficult to detect. Immunofluorescence staining for the EC marker, CD31 (Figure 3B), confirmed the observations made from SEM. Quantification and statistical analysis of EC coverage indicated that PCL/NO SDVGs significantly improved endothelial coverage at 1-month post-implantation, and there were no statistically significant differences detected in EC coverage between the two grafts after 3 months of implantation time (Figure 3C). These results suggested that PCL/NO SDVGs promoted the earlier formation of an endothelium in the graft lumen, compared to PCL SDVGs.

### Nitrate-Functionalized Grafts Improve the Formation of an Organized VSMC Layer

**FIGURE 4:**
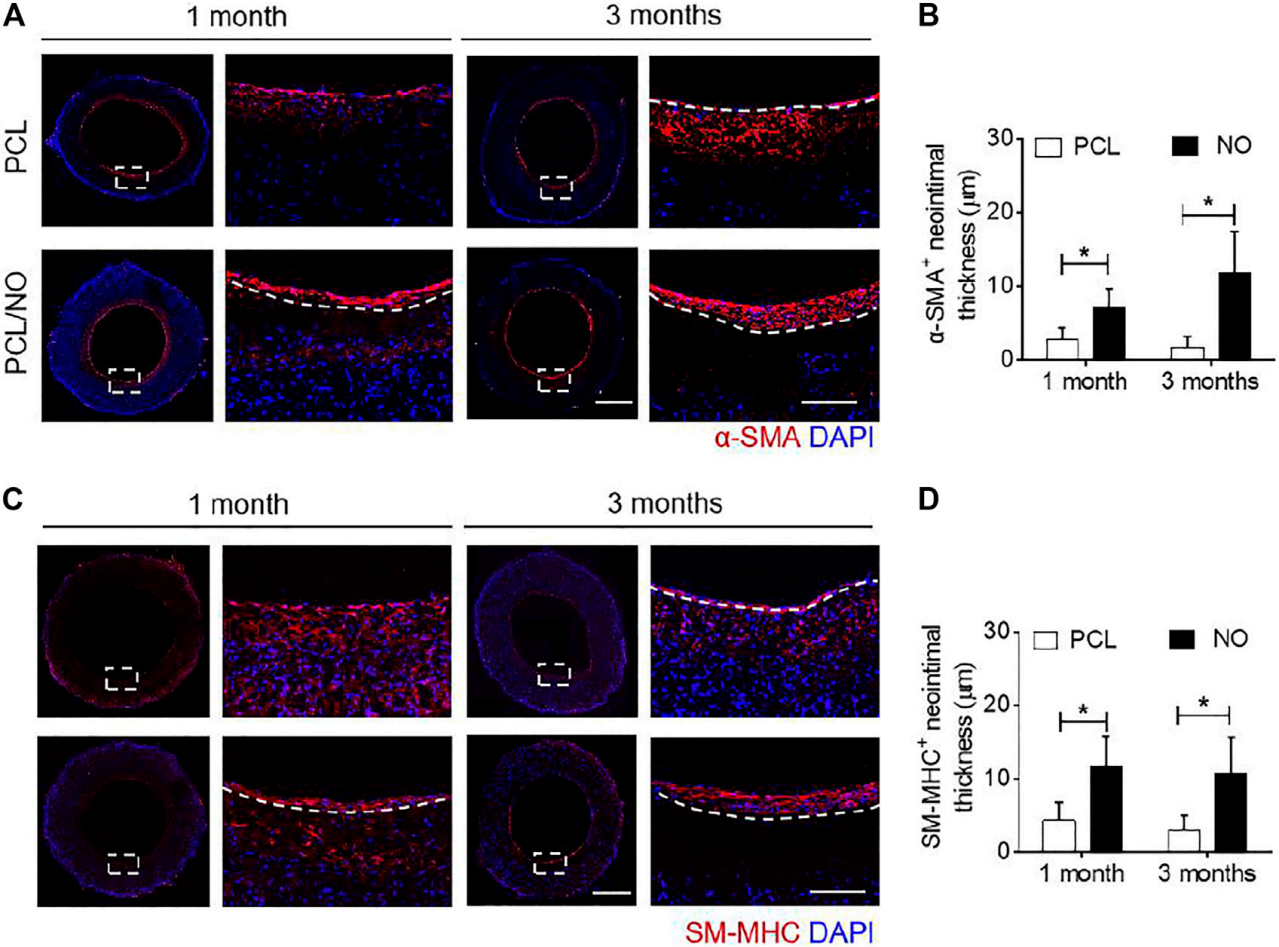
Sustained NO release from nitrate-functionalized SDVGs enhances the formation of an organized VSMC layer. **(A)** Immunohistochemical staining of SDVG enface sections (**left panels**, scale bar = 1 mm; **right panels**, scale bar = 100 µm) for α-SMA expression at 1- and 3-months post-implantation. **(B)** Quantification of average α-SMA^+^ neointimal thickness. **(C)** Immunohistochemical staining of SDVG enface sections (**left panels**, scale bar = 1 mm; **right panels**, scale bar = 100 µm) for SM-MHC expression at 1- and 3-months post-implantation. **(D)** Quantification of average SM-MHC^+^ neointimal layer thickness. Images and data are representative of *n* = 4 independent experiments. White dashed line boxes indicate zoomed area. White dashed line borders indicate the graft wall.

To evaluate vascular smooth muscle regeneration, fluorescence immunohistochemistry was performed to visualize α-smooth muscle actin (α-SMA) expressing VSMCs (Figure 4A). At 1 month, a layer of uniformly distributed α-SMA^+^ cells formed in the PCL/NO group, whereas a thin layer of α-SMA^+^ cells formed in the PCL group. At 3 months, the PCL/NO maintained a compact and uniform α-SMA^+^ cell layer. In contrast, a thin layer of α-SMA^+^ cells remained present in the PCL group and an abundance of α-SMA^+^ cells were present in the SDVG wall. The α-SMA^+^ cell layer was significantly thicker than in the control group at both time points (Figure 4B). The sections were stained to visualize smooth muscle myosin heavy chain (SM-MHC), a marker of mature and contractile VSMCs (Figure 4C). The results indicated that in the PCL group, a thin SM-MHC^+^ layer had formed but a large amount of SM-MHC^+^ cells were present throughout the SDVG wall at 1-month post-implantation. At 3 months, the PCL group displayed a more uniform layer of SM-MHC^+^ VSMCs, but the layer remained thin. In contrast, the PCL/NO group exhibited thicker uniform layers of SM-MHC^+^ cells at both 1- and 3-months post-implantation. The SM-MHC^+^ VSMCs in the PCL/NO group showed superior thickness to the PCL group (Figures 4D) and similarities in thickness and organisation to the α-SMA^+^ cell layer at 3 months, suggesting that the VSMCs in the neo-tissue possessed the contractile phenotype. These results demonstrated that NO release from PCL SDVGs enhanced the regeneration of contractile VSMC layers.

### Nitrate-Functionalized Grafts Promote Sca-1^+^ VPC Recruitment and Contribution to Vascular Regeneration

**FIGURE 5:**
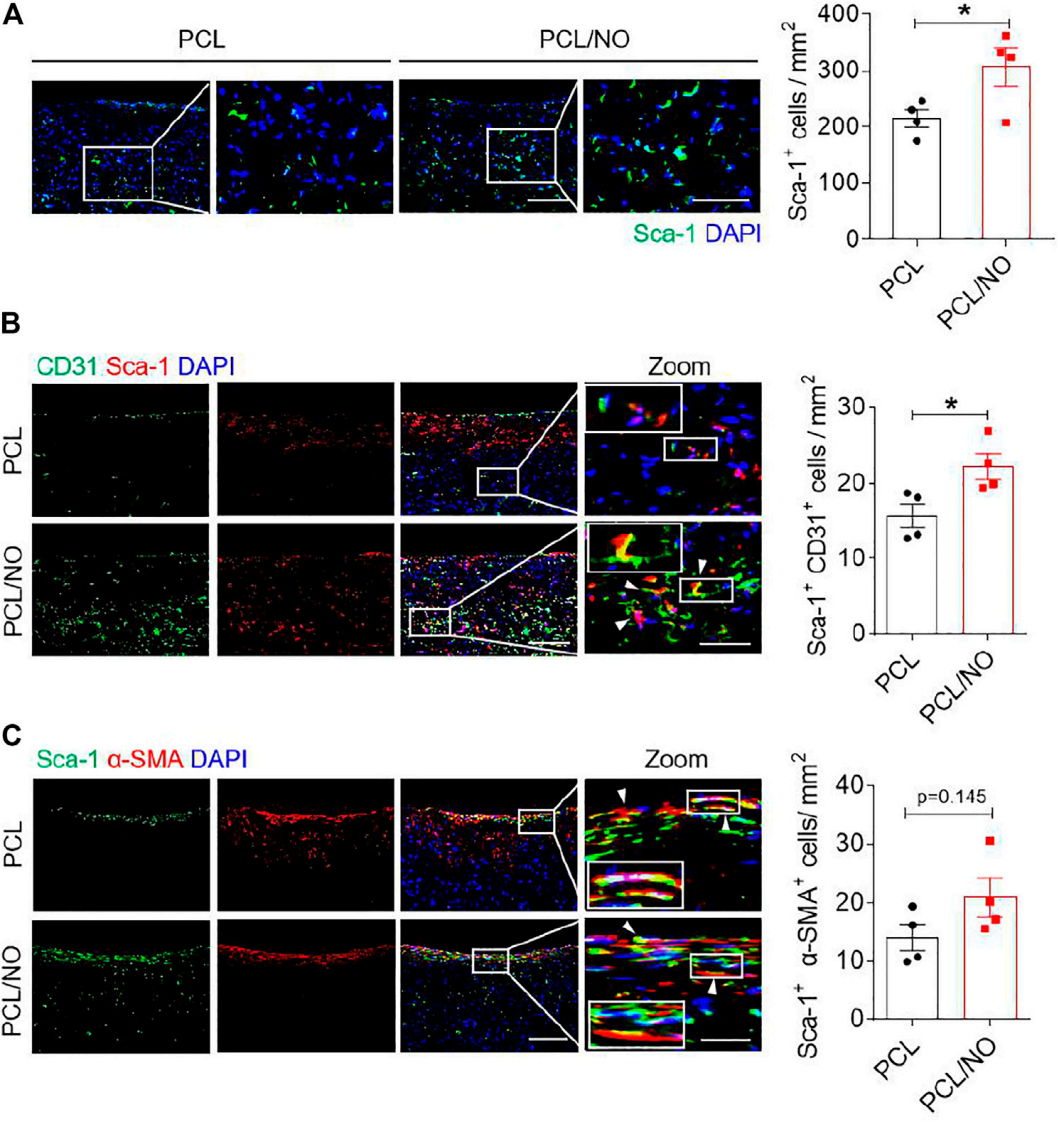
Sustained NO release from nitrate-functionalized SDVGs promotes Sca-1^+^ VPC differentiation into EC and VSMCs. **(A)** Immunohistochemical staining for Sca-1^+^ VPCs in SDVGs. Scale bar = 100 µm. The quantification of Sca-1^+^ VPCs per 1 mm^2^ section area is shown alongside. **(B)** Immunohistochemical staining for Sca-1^+^ (red)/CD31^+^ (green) ECs in SDVGs after 3 months. Scale bar = 100 µm. The quantification of Sca-1^+^/CD31^+^ ECs per 1 mm^2^ area at 3 months post-implantation is shown alongside. **(C)** Immunohistochemical staining for Sca-1^+^ (green)/α-SMA^+^ (red) VSMCs in SDVGs at 3 months post-implantation. Scale bar = 100 µm. White arrow heads indicate double-positive staining in cells. White boxes indicate the position of the inset zoomed images. The quantification of Sca-1^+^/α-SMA^+^ VSMCs per 1 mm^2^ area at 3 months post-implantation is shown alongside. Images and data are representative of *n* = 4 independent experiments.

**FIGURE 6:**
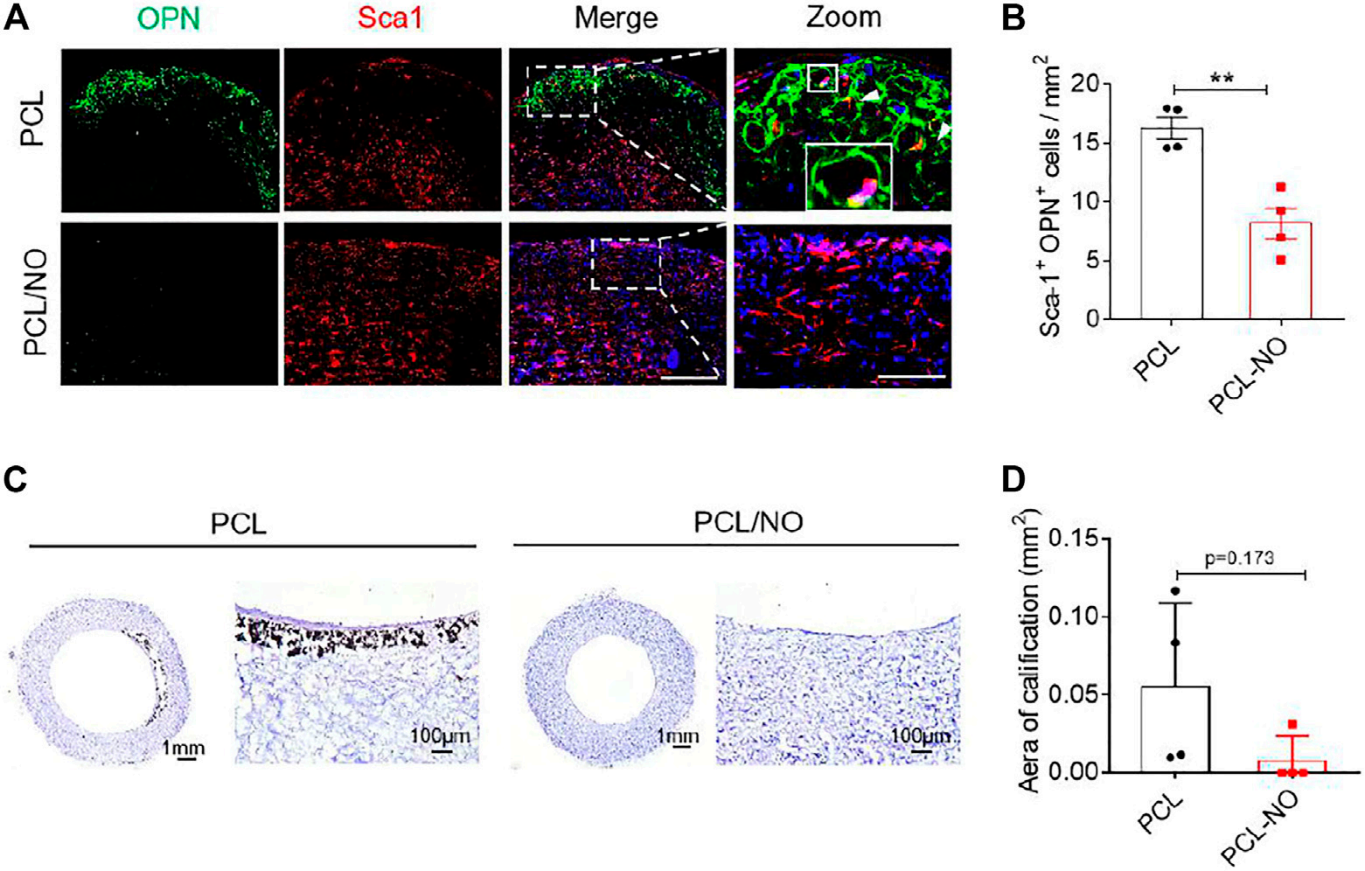
Sustained NO release from nitrate-functionalized SDVGs inhibits vascular calcification. **(A)** Immunohistochemical staining of cross sections (scale bar = 100 µm) for OPN (osteogenic cells, green) and Sca-1 (vascular progenitor cells, red). White boxes indicate area of magnified images (scale bar = 20 µm), white box shows area of inset enlarged image of positive staining around a PCL microfiber. **(B)** Quantification of Sca-1^+^/OPN^+^ cells per 1 mm^2^ area at 3 months post-implantation. **(C)** Histochemical staining of SDVG (left panels, scale bar = 1 mm; right panels, scale bar = 100 µm) sections with Von Kossa stain to visualize calcified plaque formation. **(D)** Quantification of the area of calcification present in SDVG sections at 3 months post-implantation. Images and data are representative of *n* = 4 independent experiments.

The effect of NO release on the recruitment of Sca-1^+^ VPCs was assessed at 3-months post-implantation by immunohistochemistry (Figure 5A). Infiltration of Sca-1^+^ VPCs into the SDVG walls was detectable in both graft groups. The release of NO enhanced the recruitment of Sca-1^+^ VPCs into the PCL/NO SDVGs, resulting in a significantly greater number of cells compared to the PCL group. Co-immunostaining for CD31 and Sca-1 demonstrated that a proportion of the Sca-1^+^ cells differentiated into ECs and contributed to the formation of the endothelium (Figure 5B). Sca-1^+^ cells co-expressing CD31 were most evident in the PCL/NO group, within the SDVG walls and within the endothelium, as evidenced by double-positive staining. Co-immunostaining for α-SMA and Sca-1 showed that a proportion of the Sca-1^+^ cells differentiated into VSMCs and contributed to the formation of the smooth muscle layer in both SDVG groups (Figure 5C). Vascular graft calcification is a common feature of pathogenesis associated with long-term implantation (de Valence et al., 2012; Jiang et al., 2017). Co-immunostaining to visualize Sca-1 and osteogenic marker osteopontin (OPN) revealed that a small proportion of Sca-1^+^ VPCs has differentiated to OPN^+^ cells in PCL SDVGs, and that there were significantly greater numbers of Sca-1^+^/OPN^+^ cells than in PCL/NO SDVGs, which were largely absent of OPN expressing cells (Figure 6A). Quantification of Sca-1^+^ VPCs co-expressing the different cell markers indicated that there were no statistically significant differences between Sca-1^+^/α-SMA^+^ VSMC numbers in PCL and PCL/NO groups. However, there were significantly more Sca-1^+^/CD31^+^ EC numbers and significantly fewer Sca-1^+^/OPN^+^ osteogenic cells in the PCL/NO group, compared to the PCL group (Figure 6B). Von Kossa staining for calcified tissues in SDVGs at 3 months post-implantation indicated that calcification was present in PCL SDVGs sections but largely absent in the majority of PCL/NO SDVGs sections (Figures 6C,D). However, quantification of calcified area was variable in the PCL SDVGs, indicating no statistically significant differences between PCL and PCL/NO SDVG groups. These results suggested that NO was vasculoprotective by inducing VPC differentiation into vascular cells and that NO may inhibit VPC differentiation to osteoblastic lineages, thereby enhancing vascular regeneration and limiting calcification.

## Discussion

Development of functional SDVGs with improved long-term outcomes remains a clinical demand. In physiological tissue microenvironments, NO is a crucial gasotransmitter and signalling molecule, which is continuously produced and released by ECs within the blood vessel lumens (de Mel et al., 2011). Previous studies have focused on developing NO-releasing or NO-generating materials (Enayati et al., 2021). The sustained release of NO was shown to markedly inhibit platelet adhesion and thrombus formation (Gkaliagkousi et al., 2009; Zhao et al., 2013). Our previous work further showed that NO release from vascular grafts improved tissue regeneration, remodelling, and physiological function (Wang et al., 2015). However, challenges associated with exogenous NO delivery include burst release, dose-dependent toxicity, and short half-life and diffusion distance of NO released for donors, which ultimately has restricted the commercialization of NO releasing materials (Panesar, 2008). In the present study, organic nitrates were utilized as NO donor compounds. It has been extensively demonstrated that organic nitrates undergo biotransformation to NO through different enzymatic pathways (Zhu et al., 2021). Therefore, we hypothesized that NO release from nitrate functionalized SDVGs would provide beneficial effects on the promotion of vascular regeneration whilst inhibiting the incidence of graft failure. Results showed that NO improved the regeneration of vascular grafts after 3 months of implantation, consistent with trends observed previously (Kabirian et al., 2019; Enayati et al., 2021). Overall, the prolonged, low-level release of NO achieved by PCL/NO SDVGs provided endothelium-mimicry to the graft lumen, whilst regulating VPC regeneration of the EC and VSMC layers, thus achieving markedly improved vascular regeneration, whilst minimizing the occurrence of pathological tissue remodelling.

Within the past 2 decades, several types of vascular stem cells, in addition to circulating progenitors, have been identified and characterized, with evidence that they are not only involved, but also play pivotal contributory roles in blood vessel remodelling and disease development (Wang et al., 2018; Zhang et al., 2018). Vascular tissue-resident stem cells, or vascular progenitor cells (VPCs), have been discovered to display the capacity to differentiate into vascular cell lineages, which may also contribute to the regenerative process post-graft implantation. Sca-1^+^ vascular progenitor cells (VPCs) can contribute to EC and VSMC populations during vascular remodelling (Torsney and Xu, 2011) and are important cell source mediating tissue regeneration of vascular grafts (Issa Bhaloo et al., 2018). In this work, we investigated the fates of Sca-1^+^ VPCs in the NO-enriched vascular tissue microenvironment. Sca-1^+^ VPCs are resident to the adventitial layer of the vessel walls with described roles in regeneration of blood vessels following vascular injury, but also to pathological remodelling (Hu et al., 2004). Genetic lineage tracing suggested that Sca-1 expressing VSMCs constituted over 40% of the VSMCs in the vessel wall during recovery of vessel injury. Under homeostasis and after wire injury, pre-existing VSMCs were the major source for VSMC expansion, but after significant loss of local VSMCs, VPCs from the adventitia appear to act as the source of new VSMCs in vascular repair and regeneration (Hu et al., 2004; Roostalu et al., 2018; Tang et al., 2020). Disruption of the elastic lamellae is suggested to be an important requirement that facilitates migration of adventitial VPCs to the media and intima (Psaltis and Simari, 2015; Zhang et al., 2018). This is likely to occur in human vascular pathologies and surgeries, such as transplant vasculopathy, vein graft arteriosclerosis and artificial vascular graft implantation. Our previous work demonstrated that the adventitial resident VPCs played a major role in the vascular graft regeneration (Issa Bhaloo et al., 2018; Pan et al., 2018), via migration toward the vessel graft and differentiation into ECs or VSMCs, respectively. Indeed, in the present study, we showed that following SDVG implantation, a population of Sca-1^+^ differentiated to ECs in the presence of sustained NO release; VSMCs regardless of NO release; and osteogenic lineages in the absence of NO release. The former is supported by our previous studies that demonstrated that Sca-1^+^/CD31^+^ cells migrated from the surrounding tissue to the midportion of the vascular grafts under favourable conditions (Pan et al., 2018). The latter may be indicative of material influence over cell infiltration and activation. Nonetheless, our study demonstrated that sustained release of NO exhibited a governance over Sca-1^+^ fate.

NO has been reported to play roles in modifying stem cell behaviours, such as survival, migration, proliferation, differentiation, and apoptosis (Midgley et al., 2020). Previously, it was demonstrated that NO signalling was important in the endothelial differentiation of embryonic stem cells (Nie et al., 2017). The relationship between NO and its influence over Sca-1+ VPC migration and fate has not previously been thoroughly investigated, especially in the context of vascular graft implantation. This study provides evidence that adds to the increasing pool of information suggestive of the importance of NO in directing Sca-1+ VPC differentiation into ECs and VSMCs during vascular regeneration. Indeed, we showed that the facilitation of local NO levels increased the number of CD31^+^ cells that co-expressed Sca-1^+^. However, there was a presence of a Sca-1^+^ VPC population that did not express CD31 or α-SMA, which raises the question about whether these cells contributed to vascular tissue regeneration in different way, whether directly or passively. Additionally, it is still unknown whether the increased recruitment of Sca-1^+^ specifically to the graft site was dependent on NO across the 3 months of implantation time assessed. Deeper exploration of the full extent of Sca-1^+^ VPC contribution, recruitment mechanisms, and relation to vascular graft success would form suitable research directions for future investigations.

Vascular calcification is a prominent contributor to cardiovascular morbidity and mortality (Paloian and Giachelli, 2014). Once considered a passive precipitative process, it is now appreciated that vascular calcification is an active cell-mediated process with striking resemblance to osteogenesis (Shroff et al., 2013). Previous studies have shown that Sca-1^+^ (Sca-1^+^/PDGFRα^+^ and Sca-1^+^/PDGFRα^−^) stem/progenitor cells exhibit osteoblastic differentiation potential (Cho et al., 2013; Kramann et al., 2016). These findings suggest that a subtype of vessel-resident progenitor cells offer a promising therapeutic target for the prevention of vascular calcification. Indeed, Sca-1^+^ VPCs were reported to contribute to osteoblastic lineages that drive the formation of calcified plaques (Cho et al., 2013). Here we found that following local generation of NO from grafts, numbers of Sca-1^+^/OPN^+^ cells were decreased in the vascular wall, which implied that NO inhibit vascular grafts calcification via a modulation of Sca-1^+^ VPC behaviour. The detailed molecular mechanisms of this action remained to be elucidated.

Long-term evaluation beyond the point of complete PCL degradation is rarely performed in pre-clinical studies, and this represents a key area of the research field that is lacking strong evidence in the favour of clinical adoption of PCL vascular grafts. Classic approaches to arterial substitutes prefer the use of mechanically strong materials (Niklason et al., 1999). However, biodegradability also offers an advantage for tissue remodelling and regeneration (Wu et al., 2012). Ideally, graft materials should degrade gradually in tandem with the synthesis of new ECM by the graft-populating cells, eventually resulting in a completely regenerated and polymer-free artery. Electrospun PCL vascular grafts are attractive candidates for off-the-shelf and readily available artificial vascular grafts, especially in terms of maintained patency, mechanical properties, and rapid endothelialization (Pektok et al., 2008), but PCL does have shortcomings, such as widely reported insufficiencies in facilitation of late-stage tissue remodelling and regeneration of the vascular wall, often leading to hyperplasia and calcification (de Valence et al., 2012). Therefore, integration of additional biological cues into the PCL fiber structure have formed a major focus in the development of artificial vascular grafts, and ultimately aims to achieve clinical success through the *in-situ* tissue engineering of arterial tissues. Here, we identified that NO release served as a beneficial cue for tissue regeneration in rats. However, there remains a requirement to perform extended studies in future investigations to address whether PCL/NO grafts regenerate structural and functional arterial tissue with the capability to resist arterial pressure and maintain long-term patency, beyond the point of complete PCL microfiber degradation [estimated to take 18–24 months (de Valence et al., 2012)]. Indeed, larger animal models become a necessity when assessing performance of regenerated vascular tissues after the completion of the PCL biodegradation term.

In summary, nitrate-functionalized SDVGs were successfully developed and achieved localised delivery of NO to the transplant site. In rat abdominal aorta replacement models, transplant of PCL/NO SDVGs demonstrated therapeutic efficacy, including maintenance of vessel patency and enhanced vascular regeneration, characterized by earlier regeneration of endothelium and organised smooth muscle layers, compared to PCL SDVGs. The remarkable enhancement of vascular regeneration was NO-dependent, and the prolonged release of low levels of NO from PCL/NO SDVGs promoted regenerative mechanisms, which were demonstrated to be a result of the rapid induction of Sca-1^+^ VPCs to the graft site. Our data suggested that NO plays roles in inhibiting Sca-1^+^ cell differentiation into OPN^+^ osteogenic cells, and instead promoted Sca-1^+^ cell differentiation into ECs. The rapid re-establishment of endothelial and smooth muscle layers inhibited the occurrence of graft failure and incidence of calcified plaque formation. Taken together, our data suggests that PCL/NO SDVGs are suitable artificial alternatives to autologous vessels and strong candidates for used in surgical interventions for small diameter vessel replacement or bypass surgery.

## Data Availability

The original contributions presented in the study are included in the article/Supplementary Material, further inquiries can be directed to the corresponding authors.
